# GmPHD5 acts as an important regulator for crosstalk between histone H3K4 di-methylation and H3K14 acetylation in response to salinity stress in soybean

**DOI:** 10.1186/1471-2229-11-178

**Published:** 2011-12-15

**Authors:** Tao Wu, Er-Xu Pi, Sau-Na Tsai, Hon-Ming Lam, Sai-Ming Sun, Yiu Wa Kwan, Sai-Ming Ngai

**Affiliations:** 1 Department of Biology and State (China) Key Laboratory of Agrobiotechnology, The Chinese University of Hong Kong, Shatin, Hong Kong, PR China; 2 School of Biomedical Sciences, The Chinese University of Hong Kong, Shatin, Hong Kong, PR China

## Abstract

**Background:**

Accumulated evidence suggest that specific patterns of histone posttranslational modifications (PTMs) and their crosstalks may determine transcriptional outcomes. However, the regulatory mechanisms of these "histone codes" in plants remain largely unknown.

**Results:**

In this study, we demonstrate for the first time that a salinity stress inducible PHD (plant homeodomain) finger domain containing protein GmPHD5 can read the "histone code" underlying the methylated H3K4. GmPHD5 interacts with other DNA binding proteins, including GmGNAT1 (an acetyl transferase), GmElongin A (a transcription elongation factor) and GmISWI (a chromatin remodeling protein). Our results suggest that GmPHD5 can recognize specific histone methylated H3K4, with preference to di-methylated H3K4. Here, we illustrate that the interaction between GmPHD5 and GmGNAT1 is regulated by the self-acetylation of GmGNAT1, which can also acetylate histone H3. GmGNAT1 exhibits a preference toward acetylated histone H3K14. These results suggest a histone crosstalk between methylated H3K4 and acetylated H3K14. Consistent to its putative roles in gene regulation under salinity stress, we showed that GmPHD5 can bind to the promoters of some confirmed salinity inducible genes in soybean.

**Conclusion:**

Here, we propose a model suggesting that the nuclear protein GmPHD5 is capable of regulating the crosstalk between histone methylation and histone acetylation of different lysine residues. Nevertheless, GmPHD5 could also recruit chromatin remodeling factors and transcription factors of salt stress inducible genes to regulate their expression in response to salinity stress.

## Background

Previous studies demonstrated that histone modifications such as H3 and H4 acetylation and H3S10 phosphorylation are involved in plant salinity stress [1]. Chromatin immuno-precipitation (ChIP) studies indicated that the levels of H3K4me3, H3K9ac, H3K14ac, H3K23ac and H3K27ac are altered in the coding regions of drought stress-responsive genes, including RD29A (*R*esponsive-to-*D*essication protein 29A), RD29B (*R*esponsive-to-*D*essication protein 29A), and RD20 (*R*esponsive-to-*D*essication protein 20), when they were activated under drought stress conditions [2]. Besides, the protein profile analysis of salt-responsive proteins suggests that salinity tolerance could be partially controlled by glutathione S-transferase which plays a key role in antioxidant defense mechanisms [3]. However, the detailed molecular mechanisms in these processes remain elusive.

It has been proposed that nuclear proteins can read the histone code via their PHD finger domain in HeLaS3 cells [4]. For example, the PHD finger containing protein TFIID can selectively anchor to nucleosomes by H3K4me3 [5]. Methylated H3K4 is widely considered as a marker of actively transcribing genes due to its ability to recruit other nuclear proteins [4]. In plants, PHD finger domain containing proteins may be involved in different physiological processes such vernalization-mediated epigenetic silencing and regulation of the flowering time in *Arabidopsis thaliana *[6-9]. Other PHD finger domain containing proteins, such as ORC1 (the large subunit of the origin recognition complex) can bind to H3K4me3 to regulate the origin of replication and the transcription process in *A. thaliana *[10].

There is also evidence supporting the close relationship between PHD finger domain containing proteins and salinity stress. The PHD fingers of the Alfin-like proteins in *A. thaliana *can bind to histone H3K4me3/2 [9] and the expression of the alfalfa *Aflin1 *and *Alfin1*-like (AL) genes are induced under salinity stress [11,12].

A recent investigation demonstrated that PHD homolog proteins in soybean (GmPHD) are localized in the nuclei and are up-regulated under salinity stress [13]. In the present study, we demonstrated that one of the GmPHD proteins (GmPHD5) may function as the "code reader" for methylated H3K4 in regulating the acetylated H3K14, thereby controlling the expression of targeted genes under salinity stress.

## Results

### GmPHD5 is a PHD finger domain containing protein

To elucidate the functions of PHD proteins in soybean, we obtained the full length coding region of *GmPHD5 *(see Materials and Methods) which encompasses 756 bp and encodes a protein composed of 251 amino acids (see Additional File 1, Figure S1A). SMART analysis http://smart.embl-heidelberg.de/ confirmed the presence of a PHD finger domain (with the typical C4HC3 pattern) in its C terminus (see Additional File 1, Figure S1B). In addition, amino acid sequence alignment analysis (see Additional File 1, Figure S1C) indicated that the PHD finger domain of GmPHD5 also contains features related to its interaction with histone modification. It contains the conserved aromatic amino acids that are important for the PHD finger domain to recognize the histone methylated H3K4 by forming a groove [14] and the negatively charged amino acids that are important to hold the H3R2 methylation in another groove. The result is consistent with other PHD finger domain containing proteins [14].

### Expression of GmPHD5 in soybean

Antibodies against GmPHD5 were produced by immunizing rabbits with synthetic peptides (see Materials and Methods). The anti-GmPHD5 antibodies could recognize a protein with a molecular weight ~35 kD from soybean protein extracts and also the recombinant GST-GmPHD5 protein. Pre-immunization sera were used as negative controls (see Additional File 2, Figure S2). The molecular weight of GmPHD5 detected was slightly larger than the expected value (28 kD), which could be attributed to post-translational modifications. Since there is no posttranslational modification when expressed in *E. coli*, the molecular weight of the recombinant GmPHD5 was found to be 28 kD as expected (see Additional File 2, Figure S2).

**Figure 1:**
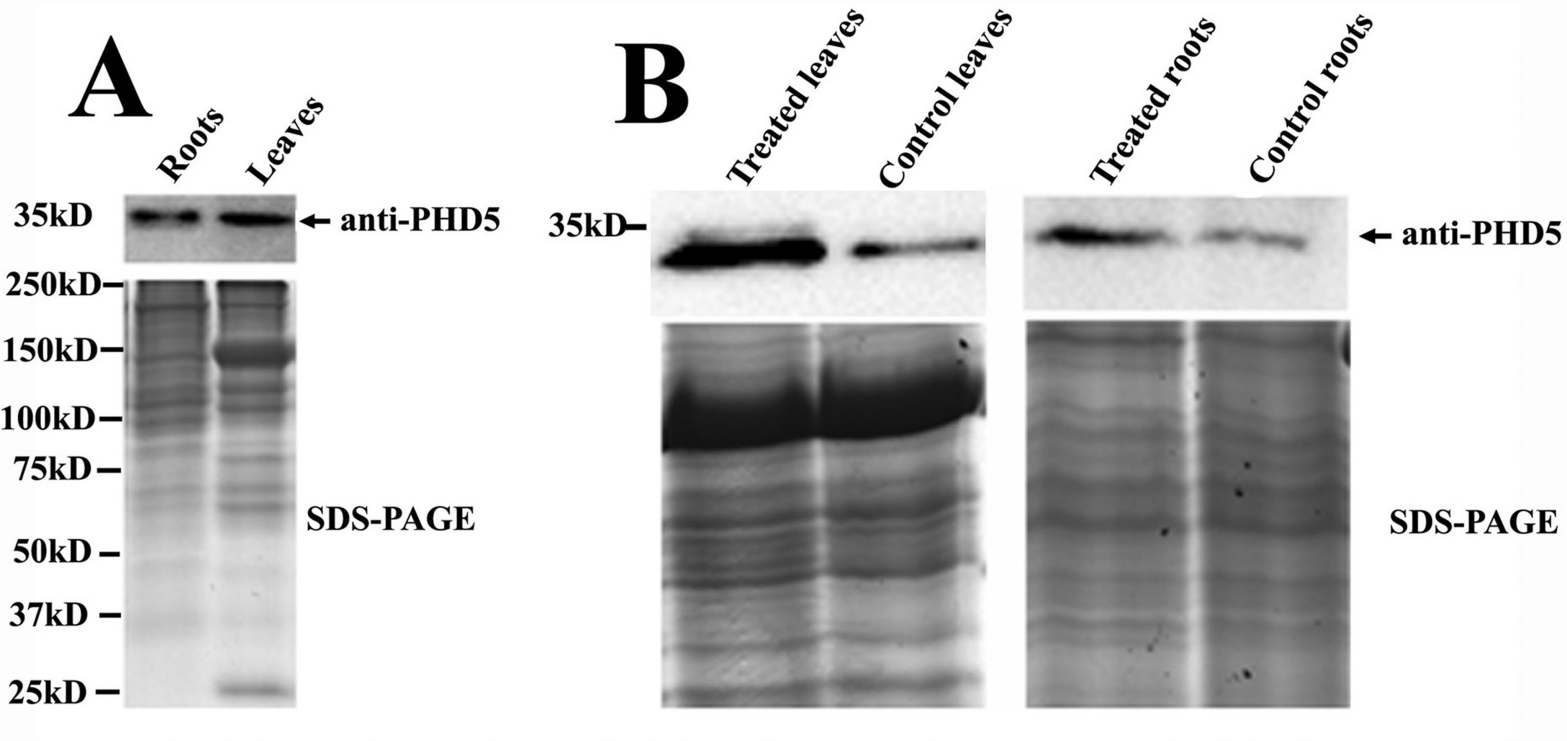
**GmPHD5 was ubiquitously expressed and its expression was up-regulated by salinity stress in soybean**. GmPHD5 was expressed in roots and leaves. Upper panel: western blotting results. Lower panel: SDS-PAGE gel image of the total proteins from soybean roots and leaves (A). GmPHD5 was upregulated by salinity stress in soybean. Upper panel: western blotting results. Lower panel: coomassie blue stained SDS-PAGE gels of soybean total proteins were showed as loading control (B).

The expression patterns of GmPHD5 in soybean were studied by western blotting. The GmPHD5 protein was found to be ubiquitously expressed in both leaves and roots (Figure 1A). In addition, the GmPHD5 protein level was found to be increased upon salinity stress in both tissues (Figure 1B).

### GmPHD5 interacts with histone methylated H3K4

**Figure 2:**
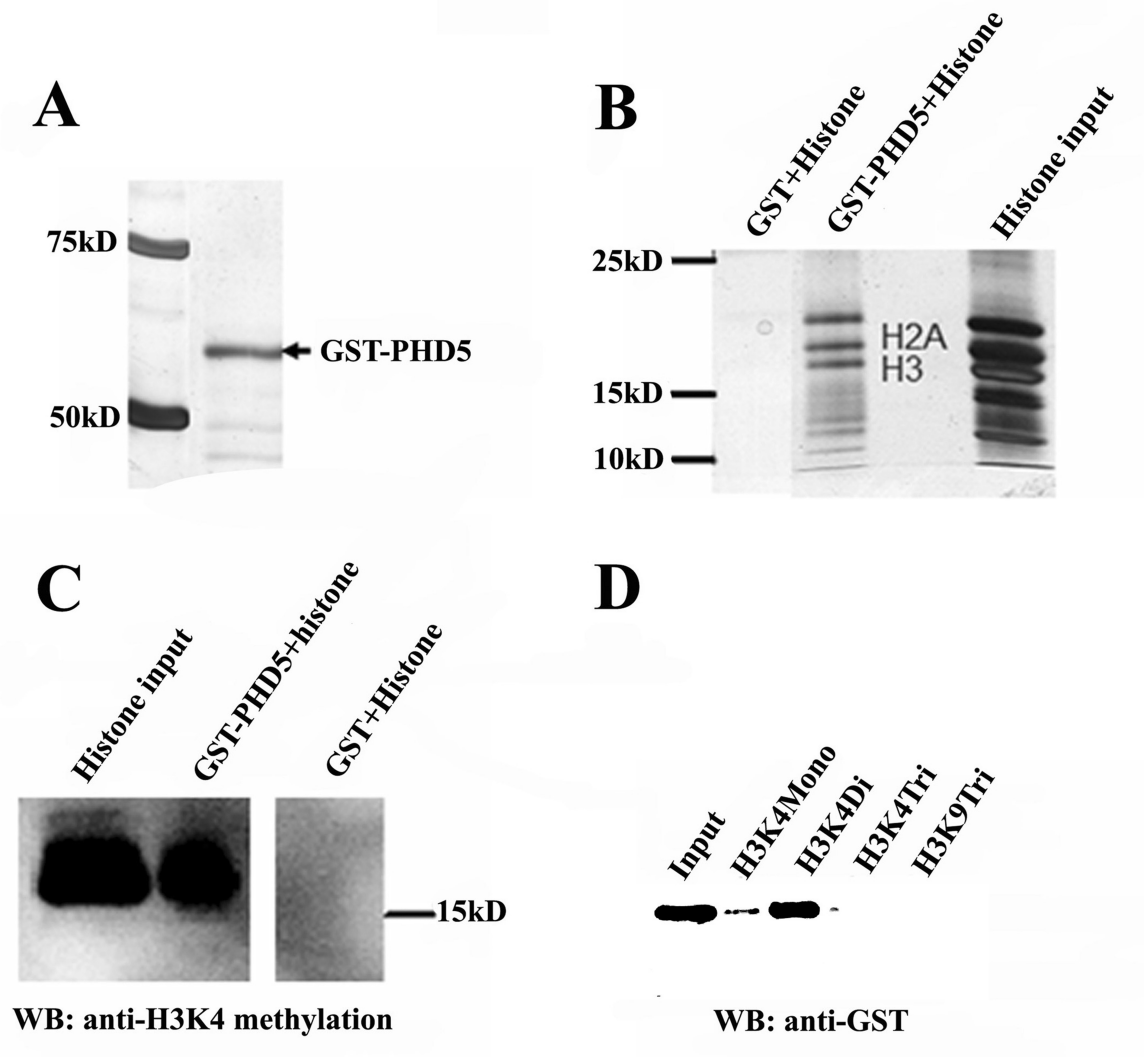
**GmPHD5 interacted with histone H3 and recognized methylated H3K4**. GST-PHD5 fusion proteins (see detail residues information in the text) were expressed in *E. coli *and purified (A). SDS-PAGE gel showed that histone H3 was pulled down by the GmPHD5 in the GST pull down assay (B). Western blotting showed that methylated H3K4 was present in the histone H3 pulled down by GmPHD5 (C). Peptide pull down assay indicated that GmPHD5 recognized methylated histone H3K4 with the preference to H3K4me2 (D).

Sequence alignment analysis with other PHD domain containing proteins suggested that GmPHD5 might interact with histone methylated H3K4 (see Additional File 1, Figure S1C). To validate our hypothesis, we expressed the GST-GmPHD5 fusion protein in *E. coli *(Figure 2A) and incubated it with histone extracted from soybean leaves. Our results clearly demonstrated that histone H3 and H2A could be co-precipitated by GST-PHD5 (Figure 2B) and methylated histone H3K4 was also confirmed in these co-precipitated histone H3 (Figure 2C).

As H3K4 can exist in mono-, di-, or tri- methylated states, we proceeded to determine the preference of GST-GmPHD5 fusion protein interaction toward these modifications. Peptide pull down assays in this study showed that GST-GmPHD5 exhibited a preferred interaction for the di-methylated H3K4 (Figure 2D). However, GST-GmPHD5 could also recognize both H3K4me and H3K4me3 with very low affinity (Figure 2D), a result that is uncommon in other PHD finger domain containing proteins such as ING protein and BPTF [15].

### Identification of non-histone proteins that interacted with GmPHD5

**Figure 3:**
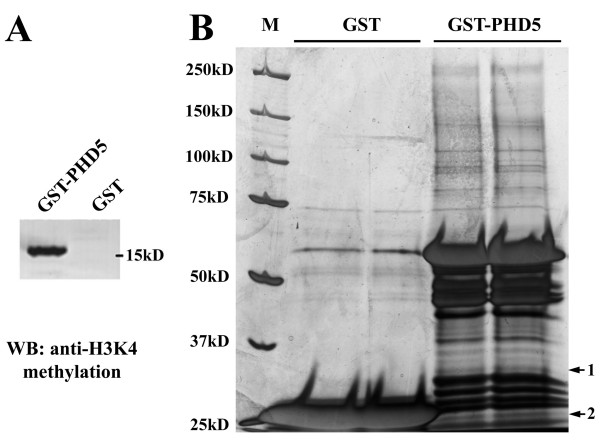
**Identification of GmPHD5 interaction proteins**. Histone H3 were pulled down by GST-PHD5 in this experiment as determined by western blotting (A). Silver staining gel of GST-PHD5 pulled down proteins. Proteins only present in the GST-PHD5 pulled down samples were picked out for mass spectrometry analysis (B). Proteins with confident identifications were indicated in the gel (band 1 and 2) (B). Protein 1 and 2 were identified as GmGNAT and GmElongin A by mass spectrometry (see Additional File 3 and 4, Figure S3 and Table S1).

We incubated the GST-GmPHD5 fusion protein with the nuclear extract from soybean to determine whether other nuclear proteins could be recruited by GmPHD5 (Figure 3B). Western blotting with anti-methylated H3K4 revealed that histone H3 was successfully pulled down (Figure 3A), validating the notion that GmPHD5 could recognize histone methylated H3K4. We subsequently identified the pulled down proteins by mass spectrometry. The identities of two non-histone proteins were successfully determined to be elongin A and GNAT (GCN5-related N-acetyltransferase family protein) (see Additional File 3 and 4, Figures S3A and S3B, and Table S1) respectively.

From the draft soybean genome, we successfully identified two isoforms of GmGNAT, namely GmGNAT1 and GmGNAT2 (GmGNAT1 is the isoform identified by mass spectrometry described above). These two isoforms display 89% homology in the nucleotide sequences of their coding region and 87% homology in their amino acid sequences, with both containing the conserved acetyltransferase domain (see Additional File 5, Figure S4). GmElongin A is a subunit of RNA polymerase II transcription factor SIII (Elongin) with a characteristic signature structure in its N terminus (see Additional File 5, Figure S4).

**Figure 4:**
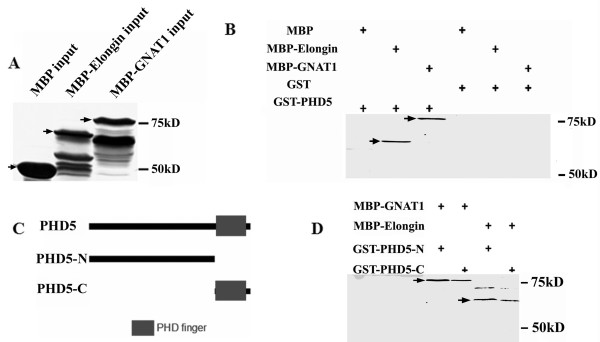
**Validation of the interaction between GmPHD5 and GmGNAT1, GmElongin A by GST pull down assay**. Inputs of the GST pull down assay. Arrowheads pointed to the expressed protein MBP (upper), MBP-Elobgin (middle) and MBP GNAT1 (lower), respectively (A). Western blotting results of the GST pull down assay. Arrowheads pointed to the pulled down protein MBP-Elongin (upper) and MBP GNAT1 (lower), respectively (B). The diagram of the construction of the truncated GmPHD5. The N termini of the GmPHD5 without its PHD finger domain and the C termini of the GmPHD5 with only the PHD finger domain were inserted into the GST expression vector (C). GST pull down assay with the truncated GmPHD5. Arrowheads pointed to the pulled down protein MBP-Elongin (upper) and MBP GNAT1 (lower), respectively (D).

The cDNA of both genes were subsequently cloned into the MBP vector to generate fusion proteins (Figure 4A). These two MBP fusion proteins were used to study the *in vitro *protein interaction towards GST-PHD5. Results showed that both MBP-GNAT1 and MBP-elongin A fusion proteins were pulled down by antibodies against GST-PHD5 (Figure 4B), which validate our previous total nuclear proteins pull down results.

To investigate which part of GmPHD5 was responsible for its interaction with GmGNAT1 and GmElongin A, truncated GmPHD5 analogs were expressed and tested *in vitro*. The N terminal polypeptide without the PHD finger domain and the C terminal polypeptide containing just the PHD finger were separately fused with GST (Figure 4C). The *in vitro *pull down assays showed that the N terminal polypeptide of GmPHD5 exhibited a stronger affinity toward GmGNAT1 than the PHD finger domain (Figure 4D). On the other hand, the truncated GmPHD5 would severely impair its interaction with GmElongin A (Figure 4D), indicating the importance of the full length of GmPHD5 in its interaction with GmElongin A.

### GmGNAT1 is an acetyltransferase

The presence of the acetyltransferase domain in the GmGNAT1 suggested that it might transfer the acetyl group to its substrates from acetyl-CoA. However, its substrates remained elusive.

**Figure 5:**
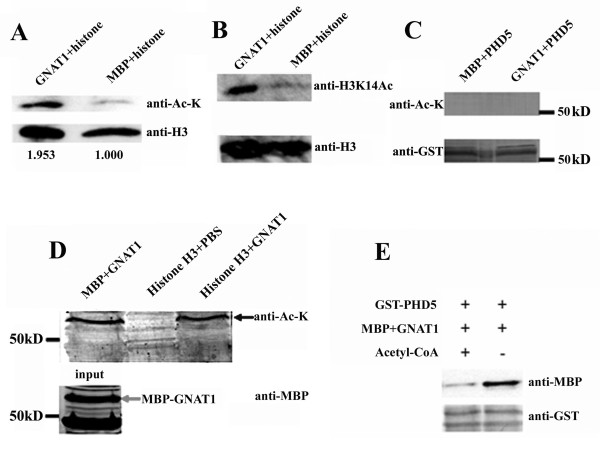
**GmGNAT1 acetylated histone H3 and itself**. *In vitro *acetyltransferase assay indicated that GmGNAT1 acetylated histone H3 (A). GmGNAT1 acetylated histone H3 mainly at histone H3K14 (B). GmGNAT1 could not acetylate GmPHD5 (C). GmGNAT1 was self-acetylated (D). GmGNAT1 self-acetylation inhibited its interaction with GmPHD5 (E).

Since GmGNAT1 interacts with GmPHD5, it might be recruited to histone H3 via GmPHD5. To test whether GmGNAT1 can acetylate GmPHD5 and the histone H3, we made use of the antibody that can specifically recognize the acetylated lysine to detect this event. Our results showed that acetylation in the extracted soybean histone H3 increased when treated with GmGNAT1 *in vitro *(Figure 5A). Since there are several adjacent lysine sites (for example, H3K9, H3K14, H3K18) in the histone H3 that are subjected to acetylation, we employed antibodies that can distinguish each site. While the extent of acetylation at histone H3 lysine 9 and lysine 18 did not change much (data not shown), the acetylated H3K14 increased significantly after treatment with GmGNAT1 (Figure 5B). However, no obvious acetylation signals were observed for GmPHD5 in a similar assay (Figure 5C), suggesting that GmPHD5 might not be the substrate of GmGNAT1 although they could interact with each other directly.

More interestingly, we found that GmGNAT1 can be self-acetylated, since the acetylation signal could be detected only when the GmGNAT1 was present (Figure 5D). Although the exact site of acetylation on GmGNAT1 is not yet known, we showed that acetylation of GmGNAT1 would impair its interaction with GmPHD5 (Figure 5E).

### GmPHD5 also interacts with GmISWI

ISWI (*I*mitation *WSI*tch) is a highly conserved protein found in yeasts to mammals [16]. ISWI contains several biologically important domains, such as DEXDx, HELICs and SANT, and functions in remodelling the chromatin structure by hydrolysing ATP. Previous reports showed that some PHD finger domain containing proteins, such as ING1 and ING2, could interact with ISWI to facilitate gene transcription [17].

**Figure 6:**
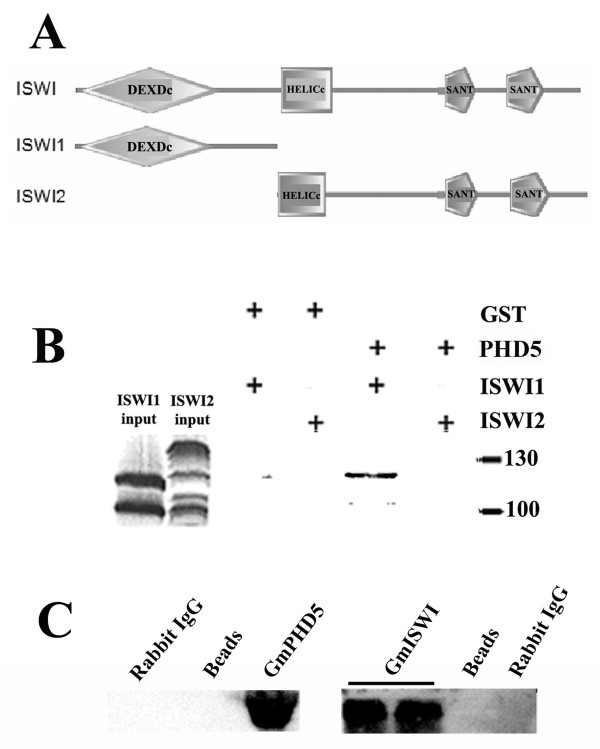
**GmPHD5 could interact with GmISWI**. The structure of GmISWI (see detail residues information in the text) and the two constructed vectors which were expressed in *E. Coli *(A). GST pull down assay indicated that GmISWI interacted with GmPHD5 through its N termini (B). The solubilized soybean nuclear proteins were immunoprecipitated with rabbit IgG, beads and anti-GmISWI antibody, respectively, followed by immunodetection using anti-GmPHD5 antibody (C). The solubilized soybean nuclear proteins were immunoprecipitated with anti-GmPHD5 antibody, rabbit IgG and beads, respectively, followed by immunodetected using anti-GmISWI antibody (C). All proteind were separated by 12% SDS-PAGE separation. These results are representative of three independent experiments.

In this investigation, we generated clones of the truncated analogs of Soybean ISWI (GmISWI): the DEXDx domain (MBP-ISWI1, residue 1 to residue 436) and the rest of GmISWI (MBP-ISWI2, residue 437 to residue 974) in *E. coli *(Figure 6A). We found that only MBP-ISWI1 but not MBP-ISWI2 could be pulled down by the GST-GmPHD5 in the GST pull down assay (Figure 6B), indicating that the N terminus of GmISWI could interact with GmPHD5.

To further confirm the interaction between GmISWI and GmPHD, we synthesized peptides of GmPHD5 and GmISWI1 to raise antibodies for co-immunoprecipitation assays (Figure 6C). When the nuclear extractions of soybean leaves were immuno-precipitated by anti-GmPHD5, GmISWI protein was detected by anti-GmISWI1 anti-GmISWI1 antibodies in the precipitant (Figure 6C). Conversely, when the anti-GmISWI1 was used to precipitate the soybean nuclear extractions in the first step, GmPHD5 domain containing proteins could be detected by anti-GmPHD5 antibodies.

### GmPHD5 located on the promoter and coding region of some salinity stress inducible genes

**Figure 7:**
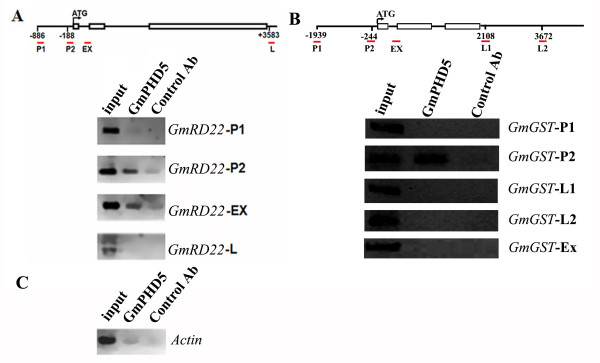
**GmPHD5 located in the promoter and body of some salt stress inducible genes**. ChIP results showed that GmPHD5 was mainly located in the near promoter region (*GmRD22*-P2) and the body (*GmRD22*-EX) of *GmRD22 *while low abundance of GmPHD5 was located in the far promoter (*GmRD22*-P1) and 3'UTR (*GmRD22*-L) of *GmRD22 *(A). GmPHD5 also located in the near promoter (*GmGST*-P2) of another salt stress inducible gene, *GmGST *(*Glycine Max Glutathione S-transferase*) (B). A very small amount of GmPHD5 located in the body of *actin*. Control Ab: preimmune antiserum (C).

Chromatin immunoprecipitation (ChIP) was performed using the anti-GmPHD5 antibody and the soybean chromatin. We detected the interaction between GmPHD5 and two salinity inducible genes (*GmRD22 *and *GmGST*; see Additional File 5, 6 and 7, Figure S4, S5 and S6). Primer sets (see Additional File 8, Table S2 and Figure 7) corresponding to the promoter and coding regions of the two salinity inducible genes (*GmRD22 *and *GmGST*) were used in this ChIP experiment. The locations of the predicted amplification regions by these primer sets are shown in Figure 7. GmPHD5 was found to bind to the promoter regions of both genes (Figure 7). For *GmRD22*, GmPHD5 also bound to its gene region near the 5' end. As negative control, GmPHD5 did not show significant binding to the actin gene nor the 3' UTR region of *GmRD22 *(Figure 7), indicating that GmPHD5 was not uniformly distributed in the genome but localized preferentially to the regulatory region of salinity stress inducible genes.

## Discussion

GmPHD5 possesses all the essential features and signatures of a PHD finger domain containing protein (see Additional File 1, Figure S1). The level of GmPHD5 increases when the plant is subjected to salinity stress (Figure 1), suggesting a functional role of GmPHD5 in stress response. This observation is consistent with a previous finding that GmPHD5 exhibits a higher expression in drought and salt-tolerant soybean accessions than the sensitive lines [13]. Incidentally, there are six homologues of PHD5 in soybean and their responses to abiotic stresses, such as salinity, cold and drought stress, are different, suggesting that although these six GmPHDs are highly conserved, their functions might be different [13]. This work showed that GmPHD5 may adopt a similar mechanism as the ING (INhibitor of Growth) family members by recruiting nuclear protein complexes to conduct the corresponding physiological functions.

GmPHD5 can interact with methylated H3K4 and exhibits amino acid sequence homology to Alfin1 in alfalfa and Alfin1-like protein in *A. thaliana*. Alfin1 is a transcriptional factor that binds to the promoter of the salt-inducible *MsPR2 *gene and enhances its expression at the transcriptional level in alfalfa roots [18]. A soybean PHD type transcription factor can also bind to the *cis*-element ''GTGGAG'' directly [13]. The methylated histone H3K4 may not initiate the interactions between GmPHD5 and its target DNA regions. However, methylated H3K4, in particular di-methylated H3K4, could definitely stabilize or enhance such interactions.

Studies indicated that the PHD finger domain could distinguish the state of lysine methylation. For example, BPTF, ING superfamily members and RAG2 mainly recognize di-, tri methylated histone H3K4, while DNMT3L and BHC80 bind to H3K4me0 [19]. However, there are apparently no findings indicating that the PHD finger proteins are able to distinguish H3K4me2 from H3K4me3. In the present study, we showed that GmPHD5 exhibits affinity to three types of histone methylated H3K4, with the following order of preference: di-methylated > mono-methylated > tri-methylated. Therefore, GmPHD5 can distinguish the subtle difference of all methylation states on H3K4.

Our present studies have also identified several non-histone proteins that can interact with GmPHD5, including GmGNAT, GmElonging A and GmISWI. GmGNAT belongs to the GNAT family, which catalyzes the transfer of an acetyl group from acetyl coenzyme A to a primary amine. For instance, the yeast GNAT selectively transfers an acetyl group to K14 of histone H3 and to K8 and K16 of histone H4 [20]. Our findings revealed that GmGNAT1 has the ability to acetylate H3K14 and possibly itself. It implies that GmGNAT together with GmPHD5 may play crucial roles in the crosstalk between histone methylation and acetylation on different amino acid residues. However, the self-acetylation pathway of GmGNAT is still unclear and a conclusive answer relies on more in depth structural analysis.

Histone acetylation is an integral part of transcriptional regulatory systems [17,21-24]. Acetylation can neutralize the positive charge of the histone and attenuate the DNA-histone contacts, resulting in the loosening of the chromatin structure to induce gene transcription [21,22]. Meanwhile, histone acetylation also affects the interaction between the amino-terminal tails and other non-histone chromatin proteins [17,23,12,25].

Our study reports a novel type of histone modification crosstalk between methylated H3K4 and acetylated H3K14, that may result in coordinating the regulation of gene transcription. To further explore the gene activation mechanism of the abovementioned histone crosstalk, it is important to identify all transcription factors and chromatin remodeling factors that can interact with GmPHD5. Our work provides evidence to show that GmPHD5 could recruit GmElongin A [26]. It is reported that the Elongin complex is a heterotrimer composed of A, B, and C subunits. Among them, the subunit A has a function in activating transcription, suggesting that GmElongin may play similar roles. In addition, GmPHD5 may also recruit the chromatin remodeling factor GmISWI which utilizes the energy from ATP hydrolysis to alter nucleosome position and/or structure.

GmPHD5 protein plays a key role in crosstalk between histone methylation and histone acetylation, recruiting gene transcription factor and chromatin remodeling factor. This implies that the histone methylation-acetylation crosstalk system (including elements such as histone PTMs, GmPHD5, GmGNAT1 and GmISWI) forms the basis of the mechanism of gene activation. For instance, since GmPHD5 is increased upon salinity stress and can interact with the promoter of selected salinity stress induced genes, such a crosstalk system might contribute to a unique transcription regulation mechanism in soybean when subjected to stress.

**Figure 8:**
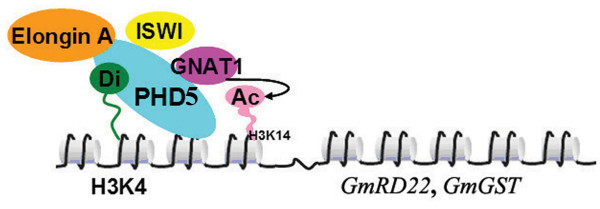
**A hypothetical model for GmPHD5 in regulating gene expression**. GmPHD5 recruited GmGNAT, GmElongin A and GmISWI to regulate gene transcription in soybean.

Our ChIP results suggest the locations of GmPHD5 in proximal region of promoter region and even within the coding region. It is in agreement with previous findings that methylated H3K4 is located in similar regions [27]. We suggest that GmPHD5 and the histone methylation-acetylation crosstalk system may be widely distributed in salinity stress inducible genes and regulate their expressions. In Figure 8, we propose a model depicting the epigenetic effects (methylated histone H3K4) on the response of soybean towards salinity stress. It seems that the histone di-methylated H3K4 of the soybean plant could be significantly increased under high salinity conditions. Subsequently, the 'histone code' H3K4me2 is recognized by GmPHD5, which is found on both the promoter and coding regions of salinity inducible genes (e.g. *GmRD22 and GmGST*, see Additional File 6 and 7, Figure S5 and S6) and may act as a regulator during activation of these genes. This regulatory complex could recruit gene expression cofactors, including the chromatin remodeling factor GmISWI, and gene transcriptional elongation factor GmElongin A. In parallel, such regulatory complex could also initiate acetylation of adjacent residues by recruiting histone acetyltransferase, and further activate salinity inducible genes.

## Conclusions

Our results demonstrate that the 'histone code' H3K4me2 could be recognized by the salinity stress inducible PHD (plant homeodomain) finger domain containing protein GmPHD5, which is found on both the promoter and coding regions of salinity inducible genes (e.g. *GmRD22 and GmGST*) and may act as a regulator during activation of these genes. Our data also leads us to propose a model for the GmPHD5 and the histone methylation-acetylation crosstalk system. We believe our investigation could definitely provide insight for the molecular basis of crosstalk between histone and other nuclear proteins within the nucleosome complex. Nonetheless, there are still many other questions to be addressed at the molecular level and further investigation is needed to address the validity of such a hypothesis.

## Methods

### Molecular cloning

Total RNA was prepared from soybean (*Glycine max *L. Merr. cv. Union) and reverse-transcribed to make the cDNA samples for molecular cloning as reported previously [28]. Gene specific primers for making cloning and the details of PCR settings were given in Additional File 9, Table S3. For sub-cloning into expression vectors, a *BamH*I (GGATCC) and a *Sal*I (GTCGAC) site were added to the 5'-ends of forward and reverse primers, respectively.

The PCR products were separated on 1% agarose gels, purified, digested with specific restriction enzymes *BamHI *or *SalI *(New England Biolabs), and sub-cloned into the expression plasmid vectors (GST: pGEX-4T-1, GE healthcare, Wisconsin, USA, Product number 28-9545-49; MBP: pMAL-C2, New England Biolabs, MA, USA) pre-digested with the same restriction enzymes. *GmPHD5 *was inserted into the GST expression vectors, while other cloned genes, *GmISWI1, GmISWI2, GmGNAT*, and *GmElongin*, were ligated into the MBP expression vector. All clones were confirmed by sequencing using the ABI PRISM™ dRhodamine Terminator Cycle Sequencing Ready Reaction kit (Perkin Elmer, Connecticut, USA; Product number: 402078) as described in the manufacturer's manual.

### Expression of recombinant proteins

Recombinant plasmids containing the target clones were transformed into the bacteria strain DE3. The transformed bacteria were inoculated into Luria-Bertani (LB) broth supplemented with 100 μg/ml of ampicillin and incubated at 37°C for 2.5-3 h until the optical density at 600 nm reached about 0.6-0.8. IPTG was then added to reach a final concentration of 1 mmol/L to induce the expression of the recombinant proteins at 25°C. After overnight expression, the bacteria were collected, suspended in phosphate buffered saline (PBS) and lysed with 1 mg/ml lysosome on ice for at least 1 h. The supernatant was collected after centrifuged at 4°C for 15 min at 21,500 g and stored at -80°C until use.

### Peptide synthesis and antibody production

Peptides (GmPHD5: GKNERKRLFQMINDLPT, residue 116 to residue 132 and TPAKAEHIKQYK, residue 230 to residue 241; GmISWI: GEEATAELDAKMKKFTEDAIK, residue 596 to residue 616) were synthesized using the standard procedures of the F-moc solid-phase peptide synthesis protocol of the Applied Biosystems 433A solid-phase peptide synthesizer. The synthesized peptides were dissolved in milli-Q water, purified by standard reversed-phase HPLC and the homogeneity of the purified peptides was determined by MALDI-TOF mass spectrometry on an ABI 4700 proteomics analyzer.

Purified peptides were conjugated to KLH (Keyhole Limpet Hemocyanin; Sigma, Missouri, USA; Product number: H8283) as described in our previous work [29]. An equal amount of complete Freunds adjuvant (Sigma, Missouri, USA; Product number: F-5881) was mixed with purified peptide-KLH solution (contain about 100 μg peptide) and emulsified manually. Six to eight-week-old rabbits were immunized with these emulsions subcutaneously. After the priming immunization, rabbits were given a booster with 100 μg antigen emulsified in incomplete Freunds adjuvant (Sigma, Missouri, USA; Product number: F-5881) (1:1) three times in two-week intervals. Finally, the serum was collected and tested by western blotting. Control serum was collected before the priming immunization. All the rabbits were raised in the animal centre of The Chinese University of Hong Kong according to animal ethics.

### Nucleic protein extraction

Soybean leaf tissue was ground into powder in liquid nitrogen, and suspended in nuclei isolation buffer (NIB) containing 20 mM Tris-HCl (pH 7.5), 10 mM KCl, 10 mM MgCl_2_, 6% sucrose, 0.6% Triton X-100, 0.05% β-mercaptoethanol, 1 mM phenylmethylsulfonyl fluoride (PMSF), as described previously [30] with some modifications. After homogenization on ice, the tissue was passed through filter paper (pore size 30 μm). The resulting nuclei fraction was harvested by centrifugation at 4000 *g *for 10 min, and then washed twice with NIB.

Isolated nuclei were swelled in low salt buffer (20 mM Tris-HCl, pH 7.6, 10 mM KCl, 2.5 mM MgCl_2_, 2 mM DTT and 0.5 mM PMSF), and total nuclear proteins were then extracted using high salt extraction buffer (500 mM NaCl, 25% glycerol in low salt buffer) [31]. The concentration of the NaCl in the extracted protein solution was diluted to 250 mM before use.

### Histone protein extraction

Nuclei isolation method followed the above nucleic protein extraction protocol [32]. The white nuclei were re-suspended in 40% guanidine hydrochloride. Then the core histones were extracted by 0.4 M HCl followed by centrifuging at 12000 g for 10 min. Finally, the core histones in the supernatant dried upon the speed vacuum system.

### Interacting between GmPHD5 and other nuclear proteins

The GST-PHD5 fusion protein was first bound to the GST column (GE healthcare, Wisconsin, USA; Product number: 17-0756-01) by incubating the protein with GST agarose beads at room temperature for 30 min. Selected nucleic proteins were then applied to the beads and incubated at 4°C overnight. The beads were washed 10 times with wash buffer (25 mM Tris-HCl (pH 8.0), 10% glycerol, 1 mM EDTA, 200 mM NaCl, 1 mM PMSF, 1 mM DTT, 0.1% Triton X-100) and subsequently boiled with SDS-PAGE gel loading buffer at 99°C for 10 min before gel separation. SDS-PAGE gels were stained with silver [32] and the target protein bands were excised, destained, and digested before subjected to MALDI-TOF/TOF for identification [32].

For the *in vitro *protein-protein interaction studies, the GST-PHD5 fusion protein was independently incubated with MBP-ISWI, MBP-ISWI2, MBP-GNAT or MBP-elongin in the GST column at 4°C overnight. Each of the equilibrated column was washed twice with the buffer containing 25 mM Tris (pH 8.0), 10% glycerol, 1 mM EDTA, 500 mM NaCl, 1 mM PMSF, 1 mM DTT, 1% Triton X-100, followed by an additional six washes with buffer containing 25 mM Tris (pH 8.0), 10% glycerol, 1 mM EDTA, 150 mM NaCl, 1 mM PMSF, 1 mM DTT, 0.1% Triton X-100. Finally, the beads from each column were separately recovered and boiled with SDS page gel loading buffer at 99°C for 10 min. Western blotting was followed as described [32] using anti-MBP antibody.

### Co-immunoprecipitation (co-IP) and peptide pull down assays

Co-immuno-precipitation (co-IP) assays were carried out using specific antibodies raised against the targeted proteins. Antibody against the biat protein was used for immuno-precipitating the complexes from the nuclear protein extracts, and the second antibody was used to detect the interacting partner via western blotting assay.

For peptide pull down assay, biotin-conjugated peptides containing H3K4me, H3K4me2 or H3K4meme3 were purchased [Millipore, Massachusetts, USA; Catalogue number: 12-563 (mono-), 12-460(di-), and 12-564(tri-)]. Biotin conjugated peptides containing H3K9 trimethylation (Millipore, Massachusetts, USA; Catalogue number: 12-568) were used as the control. The peptides were immobilized onto avidin agarose beads (Pierce, Illinois, USA; Product number: 20219). Recombinant analogs of GST-GmPHD5 was incubated with these beads at 4°C overnight. The beads were then washed twice with buffer containing 25 mM Tris buffer (pH 8.0), 10% glycerol, 1 mM EDTA, 500 mM NaCl, 1 mM PMSF, 1 mM DTT, 1% Triton X-100, followed by another six washes with buffer containing 25 mM Tris buffer (pH 8.0), 10% glycerol, 1 mM EDTA, 150 mM NaCl, 1 mM PMSF, 1 mM DTT, 0.1% Triton X-100. Finally, the beads were boiled in SDS-PAGE gel loading buffer at 99°C for 10 min and western blotting was followed using anti-GST antibody (Sigma, Missouri, USA; Product number: G7781).

### Chromatin immuno-precipitation (ChIP) assays

ChIP assays were performed using the chromatin immuno-precipitation assay kit (Millipore, Massachusetts, USA; Catalogue Number: 17-295), following the instruction in the user manual. Soybean leaves were first fixed in 1% formaldehyde for 15 min. The fixation was terminated by adding glycine to a final concentration of 125 mM. Nuclei were then extracted from the fixed leaves and re-suspended in SDS lysis buffer and incubated for 10 min on ice. The lysates were sonicated to shear the genome DNA to lengths between 200-1000 bp. Thereafter, the samples were centrifuged at 21,500 g for 10 min at 4°C. The collected supernatants were then diluted 10 fold with ChIP dilution buffer and 1% of these collected supernatants were aliquoted as input samples. The rest supernatants were subsequently pre-cleared with protein A agarose/salmon sperm DNA (50% slurry) with agitation for 1 h at 4°C. Immuno-precipitating antibody was then added and the mixture was incubated overnight with rotation at 4°C. Subsequently, protein A agarose/salmon sperm DNA (50% slurry) bead was used to precipitate the antibody/protein/DNA complexes. Then, the bead/antibody/protein/DNA complexes were washed with low salt wash buffer, high salt wash buffer, LiCl wash buffer and TE buffer sequentially. Bound protein/DNA complexes were then eluted from the beads with freshly prepared elution buffer (1% SDS, 0.1 M NaHCO_3_). To reverse the protein-DNA crosslinks, 5 M NaCl was applied to the eluted samples to a final concentration of 200 mM and heated at 65°C for over 4 h. The total DNA was finally recovered from the samples by phenol/chloroform extraction and ethanol precipitation.

ChIP-PCR reactions were set up as follows: 4 ul template (~ < 0.1 nmol) was mixed with 0.4 ul dNTP (10 mM), 0.4 ul forward primer (10 uM), 0.4 ul reverse primer (10 uM), 2 ul 10 × PCR buffer, 0.25 ul Taq polymerase (Promega, Wisconsin, USA), and 1 ul MgCl_2 _(25 mM). The final volume was adjusted to 20 ul by distilled milli-Q water. Information on primers and PCR settings were summarized in Additional File 8, Table S2. The PCR products were resolved on a 2% agarose gel.

### In vitro acetyltransferase activity assay

The MBP-GNAT recombinant protein was mixed with 125 μM Acetyl-Coenzyme A (GE healthcare, Wisconsin, USA; Product number: 27-6200-01), 60 μg histone extracted from soybean (or the other tested proteins), 1.5 mM DTT, 10% glycerol, 0.15 mM EDTA, 15 mM sodium butyl, 15 mM nicotiamide, 1 mM PMSF, 1 mM protease inhibitor, and then incubated overnight at 30°C. The reaction was then concentrated and protein acetylation was determined by western blotting with an anti-acetyl-K antibody (Millipore, Massachusetts, USA; Catalogue number: 05-515).

## Authors' contributions

PE and WT designed the study, carried out the experiment, conducted the analysis of histone modifications and drafted the manuscript. TSN carried out mass spectrometry analysis. SSM helped draft the manuscript. LHM and NSM conceived and designed the study and drafted the manuscript. All authors read and approved the final manuscript.

## Supplementary Material

Additional file 1 **Figure S1-Soybean *GmPHD5 *was a PHD finger domain containing protein**. Nucleotide and amino acid sequences of soybean *GmPHD5*. The amino acids in the red rectangle indicated the PHD finger domain (A). The GmPHD5 contained a PHD finger domain in its C terminus, which has the typical C4HC3 structure, as highlighted in pink and blue rectangles (B). Alignment of the PHD finger domain of *At*ING1, *Ms*Alfin 1, *At*AL6, *Hs*BPTF, *Hs*ING2, *Gm*PHD5. The red rectangle indicated the conserved aromatic amino acids which composed the pocket recognizing methylated H3K4. The blue rectangle indicated the conserved negative charged amino acids which composed the pocket recognizing H3R2 methylation (C). *At*ING1:at3g24010; *Ms*Alfin 1:AAA20093.2; *At*AL6: at2g02470; *Hs*BPTF: NP_872579.2; *Hs*ING2: NP_001555.1. *At: Arabidopsis thaliana*; *Ms: Medicago sativa; Hs: Homo sapiens; Gm: Glycine max*. Click here for file

Additional file 2 **Figure S2-Antibody testing of anti-PHD5 and anti-ISWI**. After solubilizing soybean leave nuclei in lysis buffer, the proteins were separated by 12% SDS-PAGE, and immunodetected with purified sera. A: The specificity of anti-PHD5 antibody was tested with recombinant GST-PHD5 by western blotting. B: The specificity of anti-PHD5 antibody was tested with soybean total proteins by western blotting. Anti-PHD5: anti-PHD5 antibody, Control: preimmune antiserum. C: The specificity of anti-ISWI antibody was tested with soybean total proteins by western blotting. Anti-ISWI: anti-ISWI antibody, Control: preimmune antiserum. Click here for file

Additional file 3 **Figure S3-Mass spectrum of protein 1 and 2 pulled down by GmPHD5**. Bands of these two proteins were manually excised out from the SDS-PAGE gel, followed by destaining and digestion procedures and then identified by MALDI-TOF/TOF. A: The mass spectrum of protein 1. B: The mass spectrum of protein 2. Click here for file

Additional file 4 **Table S1-Mass spectrometry of GNAT and Elongin A (Identified by MALDI-TOF/TOF)**. Click here for file

Additional file 5 **Figure S4-Alignment of the two GmGNATs of soybean**. These two isoforms of GmGNAT displayed 89% identities in their nucleotide sequences and 87% identities in their amino acid sequences. The GCN5-related N-acetyltransferase domain (GNAT superfamily) was indicated in this figure. Click here for file

Additional file 6 **Figure S5-Northern blot analysis GmRD22**. Soybean seeds were germinated in sand irrigated with tap water. They were then irrigated with Hoagland's solution when the first true leaves were opened. When the second trifoliates were opened, they were irrigated with Hoagland's solution supplemented with NaCl gradually increased from 0.3% to 0.6%, and finally 0.9% NaCl in 1 week interval. Control seedlings were irrigated with Hoagland's solution only. The trifoliates of each plant were collected for extraction of total RNA. Ten micrograms of total RNA was loaded onto each lane. Upper panel: Northern blot signals. Lower panel: Ethidium bromide staining of rRNA. Click here for file

Additional file 7 **Figure S6-Comparative proteomics studies demonstrating enzymes changes upon salinity stress**. The total protein of soybean whole plant were extracted by TCA/Acetone methods followed by separation using 2-DE gels procedures (A). Three proteins (glutathione S-transferase, ascorbate peroxidase and dehydroascorbate reductase), which have a well documented involvement in the glutathione-ascorbate cycle and are closely associated with ROS elimination were chosen for further validation by image analysis (B). Besides, the GmGST was also verified by Western blot analysis (C). Consistent with the observations from 2-DE analysis, expression of GmGST, MDAR and APX were up-regulated in soybean plants. Click here for file

Additional file 8 **Table S2.1-List of primers for amplifying genes GmRD22 and GmGST in Chromatin immuno-precipitation (ChIP) assays**. Table S2.2-The PCR program for amplying genes *GmRD22 *and *GmGST*. Click here for file

Additional file 9 **Table S3.1-List of primers of genes *GmPHD5, GmISWI1, GmISWI2, GmGNAT *and *GmElongin *used for expression analyses**. The sequences underlined indicated homologous regions to linear donor vector both ends. Table S3.2-The PCR program for cloning genes *GmPHD5, GmISWI1, GmISWI2, GmGNAT *and *GmElongin*. Click here for file
