# Supplementary figures and images for: GmPHD5 acts as an important regulator for crosstalk between histone H3K4 di-methylation and H3K14 acetylation in response to salinity stress in soybean
Source: BMC Plant Biol. 2011 Dec 15;11:178. doi: 10.1186/1471-2229-11-178 (PMC3288756; doi:10.1186/1471-2229-11-178)

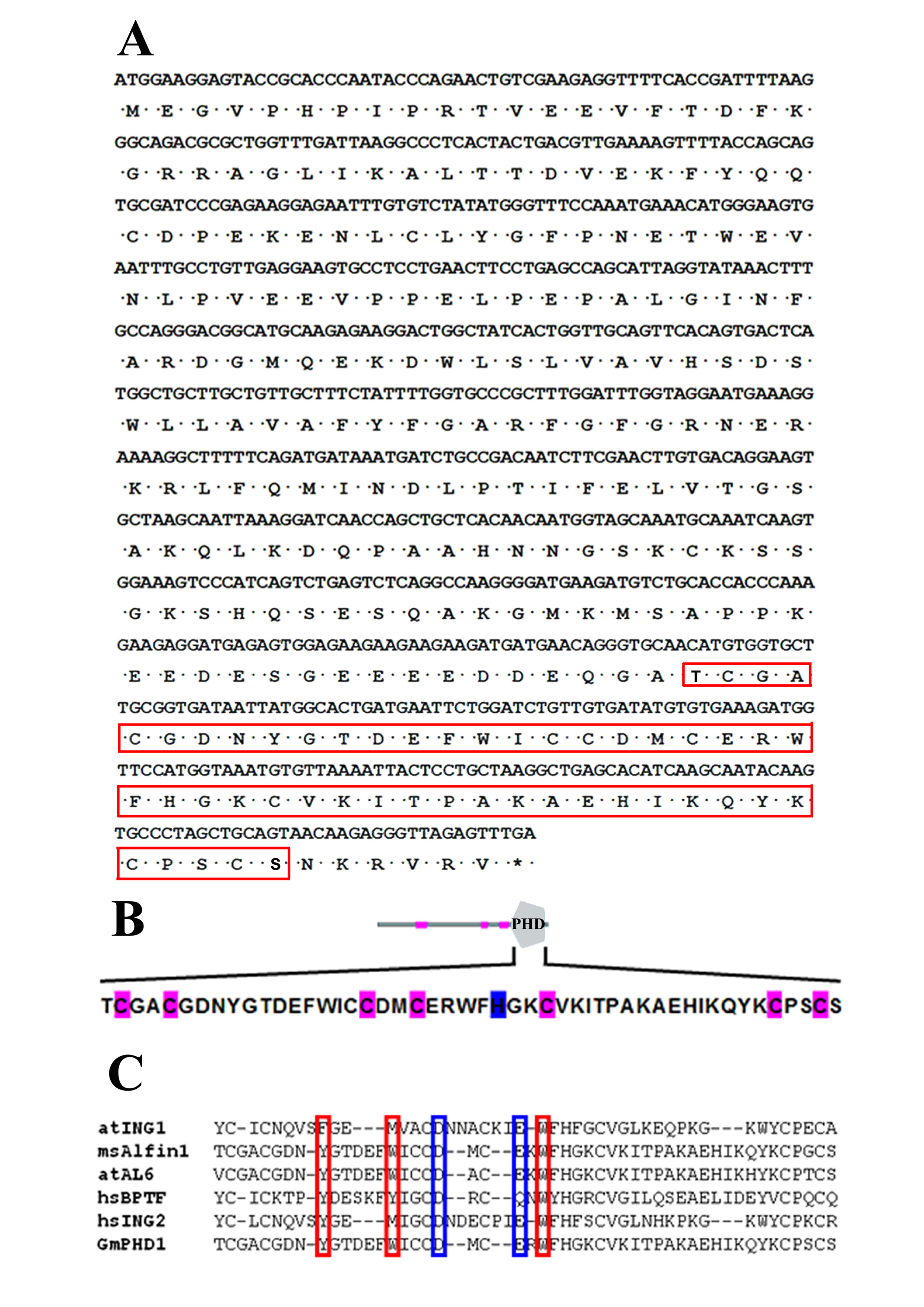

Supplement: Additional file 1 — Figure S1-Soybean GmPHD5 was a PHD finger domain containing protein. Nucleotide and amino acid sequences of soybean GmPHD5. The amino acids in the red rectangle indicated the PHD finger domain (A). The GmPHD5 contained a PHD finger domain in its C terminus, which has the typical C4HC3 structure, as highlighted in pink and blue rectangles (B). Alignment of the PHD finger domain of AtING1, MsAlfin 1, AtAL6, HsBPTF, HsING2, GmPHD5. The red rectangle indicated the conserved aromatic amino acids which composed the pocket recognizing methylated H3K4. The blue rectangle indicated the conserved negative charged amino acids which composed the pocket recognizing H3R2 methylation (C). AtING1:at3g24010; MsAlfin 1:AAA20093.2; AtAL6: at2g02470; HsBPTF: NP_872579.2; HsING2: NP_001555.1. At: Arabidopsis thaliana; Ms: Medicago sativa; Hs: Homo sapiens; Gm: Glycine max. [file 1471-2229-11-178-S1.JPEG]

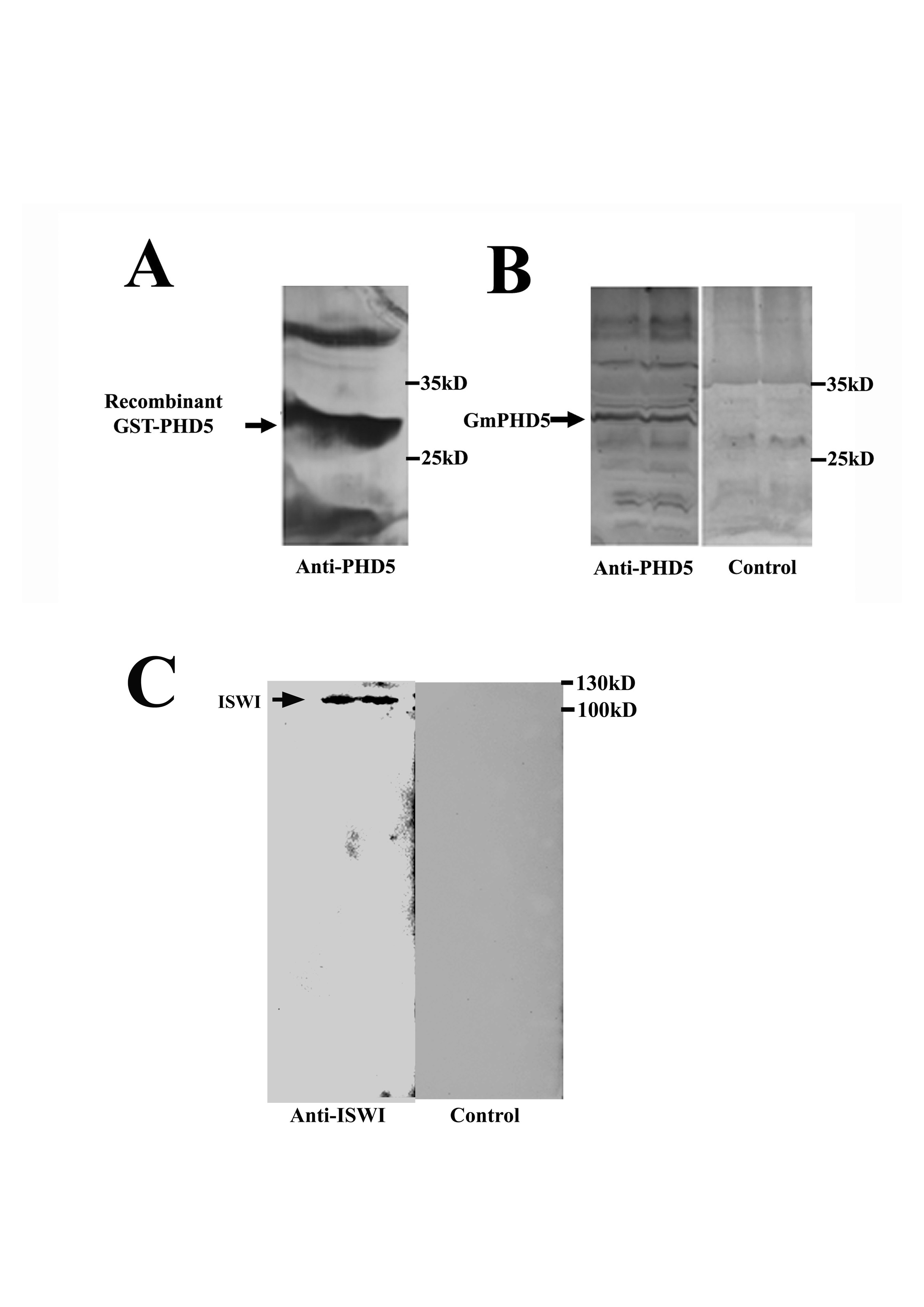

Supplement: Additional file 2 — Figure S2-Antibody testing of anti-PHD5 and anti-ISWI. After solubilizing soybean leave nuclei in lysis buffer, the proteins were separated by 12% SDS-PAGE, and immunodetected with purified sera. A: The specificity of anti-PHD5 antibody was tested with recombinant GST-PHD5 by western blotting. B: The specificity of anti-PHD5 antibody was tested with soybean total proteins by western blotting. Anti-PHD5: anti-PHD5 antibody, Control: preimmune antiserum. C: The specificity of anti-ISWI antibody was tested with soybean total proteins by western blotting. Anti-ISWI: anti-ISWI antibody, Control: preimmune antiserum. [file 1471-2229-11-178-S2.JPEG]

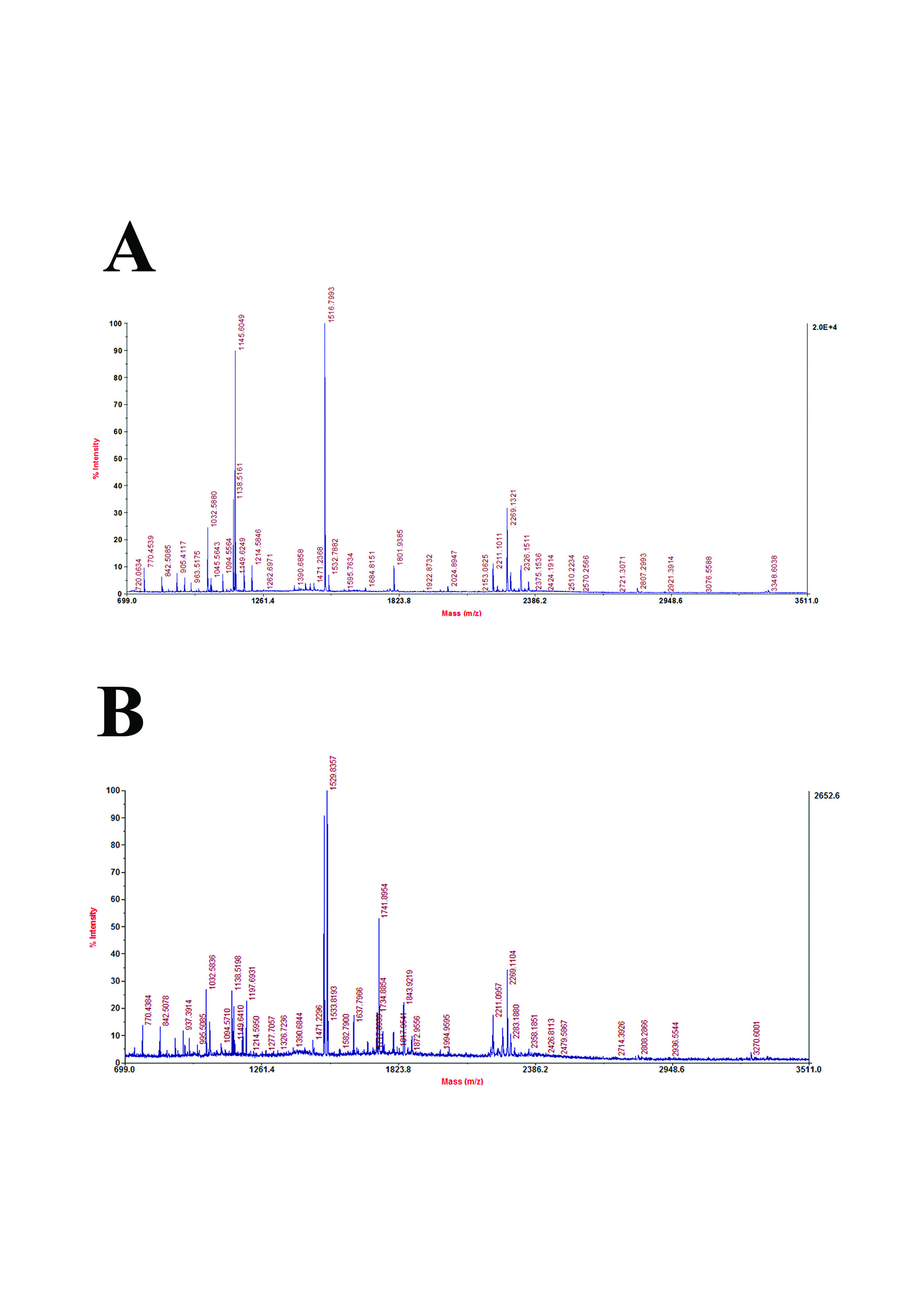

Supplement: Additional file 3 — Figure S3-Mass spectrum of protein 1 and 2 pulled down by GmPHD5. Bands of these two proteins were manually excised out from the SDS-PAGE gel, followed by destaining and digestion procedures and then identified by MALDI-TOF/TOF. A: The mass spectrum of protein 1. B: The mass spectrum of protein 2. [file 1471-2229-11-178-S3.JPEG]

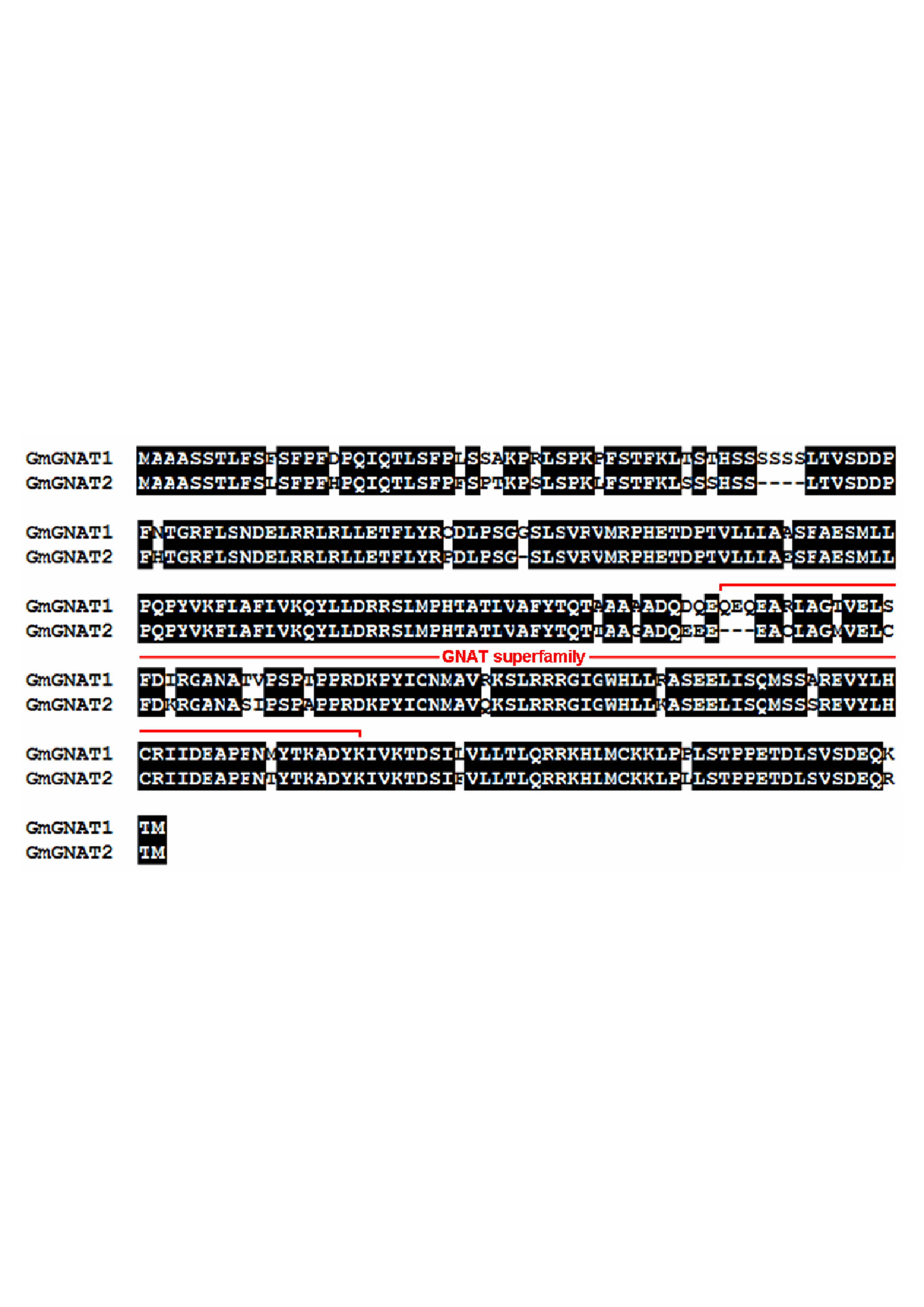

Supplement: Additional file 5 — Figure S4-Alignment of the two GmGNATs of soybean. These two isoforms of GmGNAT displayed 89% identities in their nucleotide sequences and 87% identities in their amino acid sequences. The GCN5-related N-acetyltransferase domain (GNAT superfamily) was indicated in this figure. [file 1471-2229-11-178-S5.JPEG]

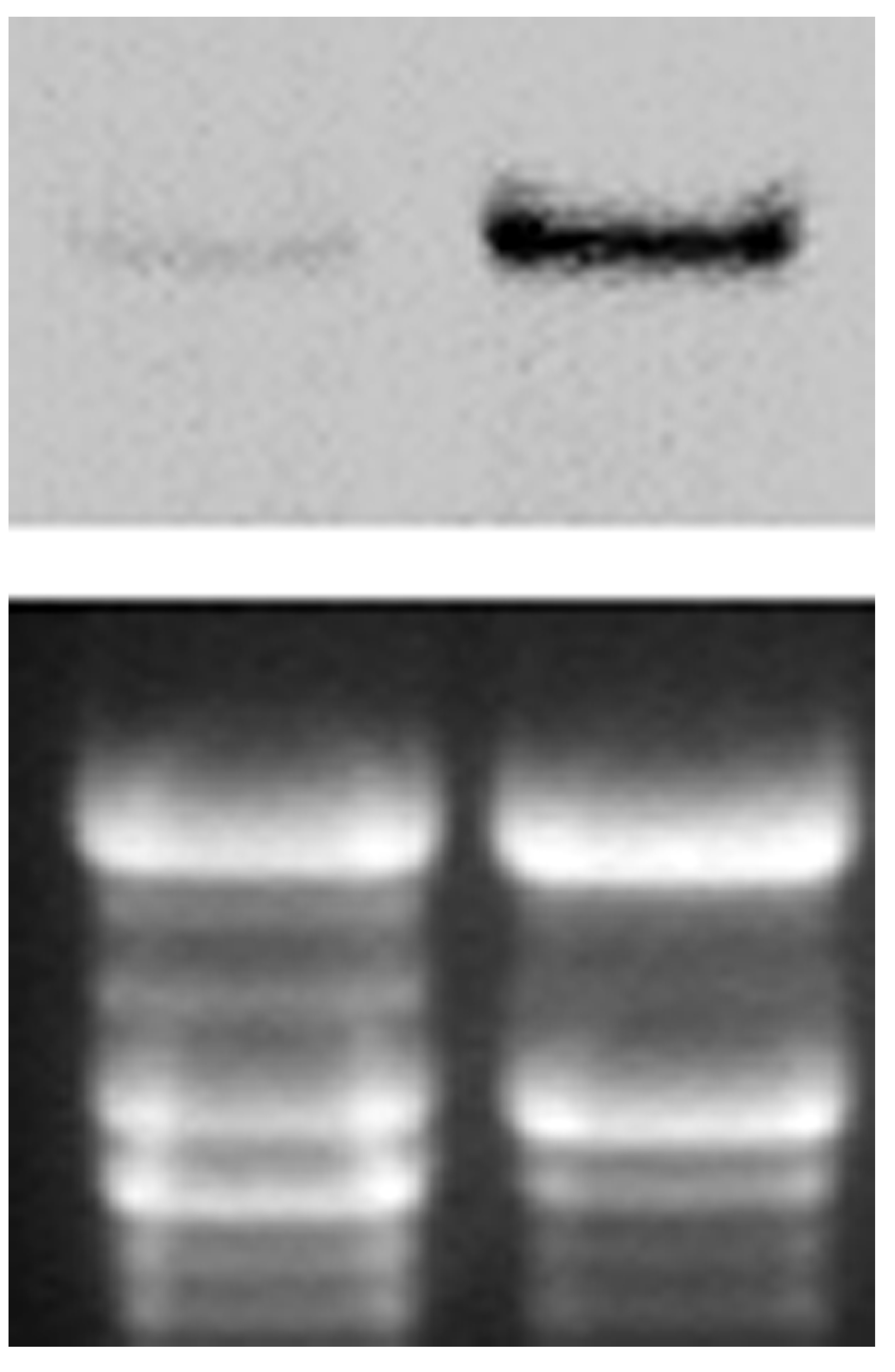

Supplement: Additional file 6 — Figure S5-Northern blot analysis GmRD22. Soybean seeds were germinated in sand irrigated with tap water. They were then irrigated with Hoagland's solution when the first true leaves were opened. When the second trifoliates were opened, they were irrigated with Hoagland's solution supplemented with NaCl gradually increased from 0.3% to 0.6%, and finally 0.9% NaCl in 1 week interval. Control seedlings were irrigated with Hoagland's solution only. The trifoliates of each plant were collected for extraction of total RNA. Ten micrograms of total RNA was loaded onto each lane. Upper panel: Northern blot signals. Lower panel: Ethidium bromide staining of rRNA. [file 1471-2229-11-178-S6.JPEG]

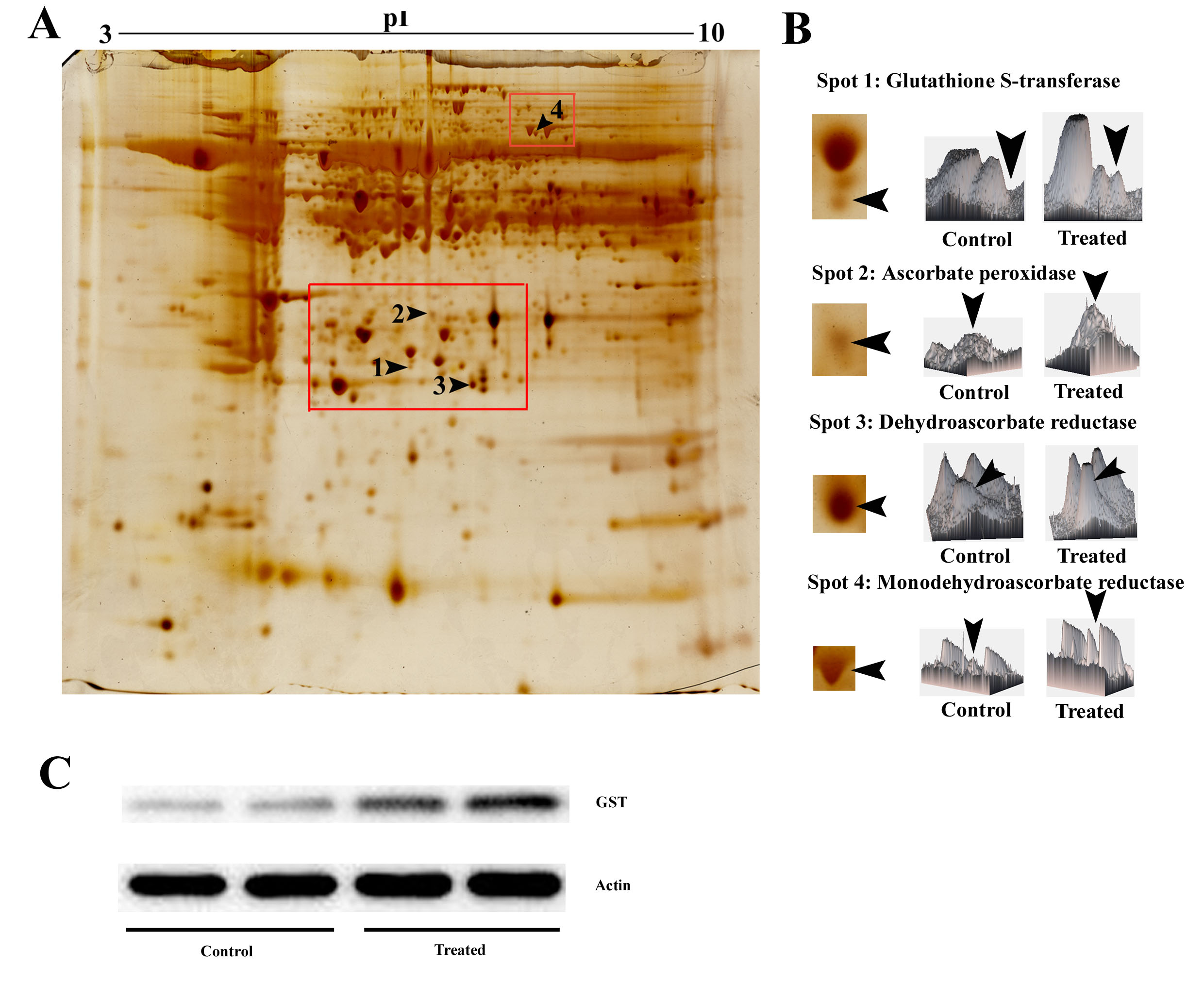

Supplement: Additional file 7 — Figure S6-Comparative proteomics studies demonstrating enzymes changes upon salinity stress. The total protein of soybean whole plant were extracted by TCA/Acetone methods followed by separation using 2-DE gels procedures (A). Three proteins (glutathione S-transferase, ascorbate peroxidase and dehydroascorbate reductase), which have a well documented involvement in the glutathione-ascorbate cycle and are closely associated with ROS elimination were chosen for further validation by image analysis (B). Besides, the GmGST was also verified by Western blot analysis (C). Consistent with the observations from 2-DE analysis, expression of GmGST, MDAR and APX were up-regulated in soybean plants. [file 1471-2229-11-178-S7.JPEG]
