# Supplementary material for: GmPHD5 acts as an important regulator for crosstalk between histone H3K4 di-methylation and H3K14 acetylation in response to salinity stress in soybean
Source: BMC Plant Biol. 2011 Dec 15;11:178. doi: 10.1186/1471-2229-11-178 (PMC3288756; doi:10.1186/1471-2229-11-178)
Supplement: Additional file 4 — Table S1-Mass spectrometry of GNAT and Elongin A (Identified by MALDI-TOF/TOF). [file 1471-2229-11-178-S4.DOC]

**Additional file 4, Table S1 - Mass spectrometry of GNAT and Elongin A (Identified by MALDI-TOF/TOF)**

|  |  |  |  |  |
| --- | --- | --- | --- | --- |
| Protein | Accession no. | Protein mass | MS | Peptide sequence |
| GNAT | XP_002531402.1 | 33781.91 | 1149.6249 | FLSNDELRR |
|  |  |  | 963.5175 | QYLLDRR |
|  |  |  | 2570.2566 | GIGWHLLRASEELISQMSSAREVYLHCRIIDEAPFNMYTK |
| Elongin A | ACU16340.1 | 26601.68 | 770.4384 | VPPSSKR |
|  |  |  | 1094.19 | TGSISGIGSTSK |
|  |  |  |  |  |
