# Supplementary material for: GmPHD5 acts as an important regulator for crosstalk between histone H3K4 di-methylation and H3K14 acetylation in response to salinity stress in soybean
Source: BMC Plant Biol. 2011 Dec 15;11:178. doi: 10.1186/1471-2229-11-178 (PMC3288756; doi:10.1186/1471-2229-11-178)
Supplement: Additional file 8 — Table S2.1-List of primers for amplifying genes GmRD22 and GmGST in Chromatin immuno-precipitation (ChIP) assays. Table S2.2-The PCR program for amplying genes GmRD22 and GmGST. [file 1471-2229-11-178-S8.DOC]

**Additional file 8, Table S2.1 - List of primers for amplifying genes GmRD22 and GmGST in Chromatin immuno-precipitation (ChIP) assays.**

| Genes | Primers | |
| --- | --- | --- |
| *GmRD22-P1* | Forward | 5’ CGTTTAACTGTTTAAGGAA 3’ |
|  | Reverse | 5’ AAATGGGTAGGAGACGAT 3’ |
| *GmRD22-P2* | Forward | 5’ GGAAAAGAAGAAAGCCACACTCTGA 3’ |
|  | Reverse | 5’ AATTGGACATTGATCGATTGATGA 3’ |
| *GmRD22-Ex* | Forward | 5’ AGGCCAGGTGCTGCATAATCT 3’ |
|  | Reverse | 5’ ATGGATTGCCACCAGTGCAA 3’ |
| *GmRD22-L* | Forward | 5’ AGTCCTTCTTTCCTGTTCT 3’ |
|  | Reverse | 5’ TACTATCTACAACGATTTAC 3’ |
| *GmGST-P1* | Forward | 5’ AGCCACATGGATTCAGGTTC 3’ |
|  | Reverse | 5’ TTCCCCTCCTGCATTAACAA 3’ |
| *GmGST-P2* | Forward | 5’ TGTTAATGCAGGAGGGGAAT 3’ |
|  | Reverse | 5’ GGCTGCAATTCACGTTCTCT 3’ |
| *GmGST-Ex* | Forward | 5’ ATGAAATTATCCCCGATAAC 3’ |
|  | Reverse | 5’ TTAATTACAATAAAAATGAATTATA 3’ |
| *GmGST-L1* | Forward | 5’ GAGGTTCAGCGAAGGAGAGA 3’ |
|  | Reverse | 5’ GTGTTTGTGCATTCGGGTTA 3’ |
| *GmGST-L2* | Forward | 5’ CAGCGATGCCAATAGTTTGA 3’ |
|  | Reverse | 5’ ATCATTGTTGGGCTTGCATA 3' |
|  |  |  |

**Additional file 8, Table S2.2 - The PCR program for amplying genes *GmRD22* and *GmGST*.**

|  |  |  |
| --- | --- | --- |
| Number of cycles | Length of time | Temperature |
| 1 cycle | 5 minutes | 94 ^o^C |
| 40 cycles | 30 seconds | 94 ^o^C |
|  | 30 seconds | 46 ^o^C |
|  | 1 minute | 72 ^o^C |
| 1 cycle | 10 minute | 72 ^o^C |
|  |  |  |
