# Supplementary material for: GmPHD5 acts as an important regulator for crosstalk between histone H3K4 di-methylation and H3K14 acetylation in response to salinity stress in soybean
Source: BMC Plant Biol. 2011 Dec 15;11:178. doi: 10.1186/1471-2229-11-178 (PMC3288756; doi:10.1186/1471-2229-11-178)
Supplement: Additional file 9 — Table S3.1-List of primers of genes GmPHD5, GmISWI1, GmISWI2, GmGNAT and GmElongin used for expression analyses. The sequences underlined indicated homologous regions to linear donor vector both ends. Table S3.2-The PCR program for cloning genes GmPHD5, GmISWI1, GmISWI2, GmGNAT and GmElongin. [file 1471-2229-11-178-S9.DOC]

| **Additional file 9, Table S3.1 - List of primers of genes *GmPHD5*, *GmISWI1*, *GmISWI2*, *GmGNAT* and *GmElongin* used for expression analyses. The sequences underlined indicated homologous regions to linear donor vector both ends.** | | |
| --- | --- | --- |
| Genes | Primers | |
| *GmPHD5* | Forward | 5’ AGTGGATCCGAAGGAGTACCGCACCCAA 3’ |
|  | Reverse | 5’ TCAGTCGACCTCAAACTCTAACCCTCTTGT 3’ |
| *GmISWI1* | Forward | 5’ AGTGGATCCCAGAAAATGAAGAAACAGAAG 3’ |
|  | Reverse | 5’ TCAGTCGACCTCATAAATAATCTTCGAGTATGTC 3’ |
| *GmISWI2* | Forward | 5’ AGTGGATCCATGTTTCGTGGATATCAAT 3’ |
|  | Reverse | 5’ TCAGTCGACCTTATTTTCTTCTCTTCCCC 3’ |
| *GmGNAT* | Forward | 5’ AGTGGATCCATGGCTGCAGCATCATCAA 3’ |
|  | Reverse | 5’ TCAGTCGACCTCACATAGTCTTTTGCTCAT 3’ |
| *GmElongin* | Forward | 5’ AGTGGATCCATGATGAGAAGAGATCAA 3’ |
|  | Reverse | 5’ TCAGTCGACCCTAAAATACCCTCTTTGT 3’ |
| *GmPHD5-C* | Forward | 5’ AGTGGATCCACATGTGGTGCTTGCGGTG 3’ |
|  | Reverse | 5’ TCAGTCGACCTCAAACTCTAACCCTCTTGT 3’ |
| *GmPHD5-N* | Forward | 5’ AGTGGATCCGAAGGAGTACCGCACCCAA 3’ |
|  | Reverse | 5' TCAGTCGACCTCATGCACCCTGTTCATCATCT 3' |
|  |  |  |
|  |  |  |
| **Additional file 9, Table S3.2 - The PCR program for cloning genes *GmPHD5*, *GmISWI1*, *GmISWI2*, *GmGNAT* and *GmElongin*.** | | |
|  |  |  |
| Number of cycles | Length of time | Temperature |
| 1 cycle | 5 minutes | 94 ^o^C |
| 5 cycles | 30 seconds | 94 ^o^C |
|  | 30 seconds | 45 ^o^C |
|  | 1 minute | 72 ^o^C |
| 25 cycles | 30 seconds | 94 ^o^C |
|  | 30 seconds | 50 ^o^C |
|  | 1 minute | 72 ^o^C |
| 1 cycle | 10 minutes | 72 ^o^C |
